# Protocol for screening facultative parthenogenesis in *Drosophila*

**DOI:** 10.1016/j.xpro.2023.102585

**Published:** 2023-09-22

**Authors:** Alexis L. Sperling, David M. Glover

**Affiliations:** 1 Department of Genetics, University of Cambridge, Cambridge, CB2 3EH Cambridgeshire, UK; 2 Division of Biology and Biological Engineering, California Institute of Technology, Pasadena, CA 91125, USA

**Keywords:** Genetics, Evolutionary Biology, Model Organisms

## Abstract

Most species of sexually reproducing *Drosophila* are capable of some degree of facultative parthenogenesis, which involves the initiation of development in an unfertilized egg. Here, we present an optimized protocol to screen facultative parthenogenesis in *Drosophila*. We describe steps for the collection and maintenance of virgin flies. We then detail offspring screening for the analysis of parthenogenesis. This protocol can be applied to different *Drosophila* strains and can be adapted for the analysis of parthenogenesis in other animals.

For complete details on the use and execution of this protocol, please refer to Sperling et al.^1^

## Highlights

• A protocol for parthenogenesis screening in *Drosophila*
• Screening genetic variants in *Drosophila melanogaster* for parthenogenesis
• Temperature optimization for parthenogenesis screening
• Control selection for parthenogenesis screening

## Abstract (editor highlights)

Publisher’s note: Undertaking any experimental protocol requires adherence to local institutional guidelines for laboratory safety and ethics.

## Graphical abstract

**Figure:**
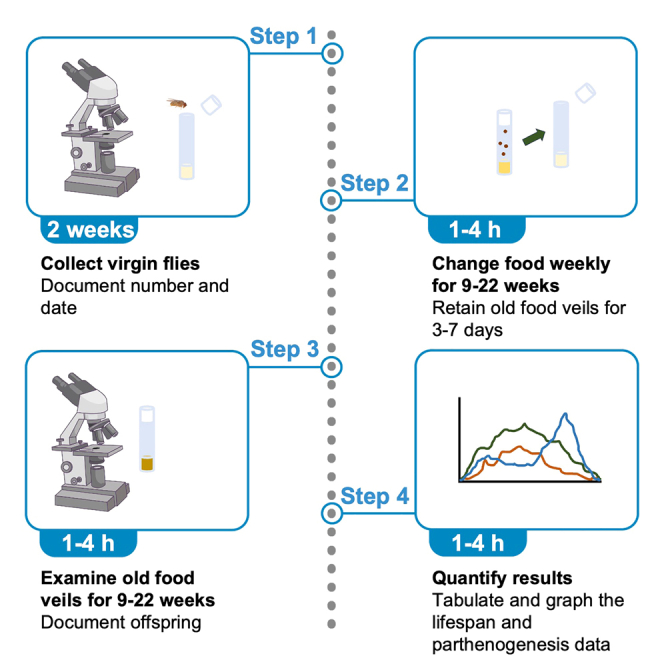

## Before you begin

### Preparation Step 1: Determining the baseline of parthenogenesis in the genus

If screening a species from a genus for which facultative parthenogenesis has not been as extensively studied as *Drosophila*,^2^^,^^3^^,^^4^ we recommend performing preliminary screens with multiple different species from that genus, if possible, to get a baseline and determine a potential range of parthenogenetic ability. When developing this screening method, we screened 13 different *Drosophila* species in order to first determine a baseline indication of facultative parthenogenesis in *Drosophila*. We determined that testing the ability of approximately 500 virgin female flies to generate progeny was sufficient for *Drosophila* to identify low levels (1 offspring per 500 females screened) of parthenogenesis. High levels (3 offspring per female screened) of parthenogenesis could be reliably detected with as few as 30 flies.^1^ We also suggest screening different strains of the same species since facultative parthenogenesis does appear to be strain and geographical collection point dependent.^3^^,^^5^^,^^6^^,^^7^ We assessed three different strains of *Drosophila melanogaster* and found that two typical laboratory strains of *D. melanogaster* (*w*^*-*^ and Oregon-R) showed no parthenogenesis whatsoever, whereas a strain caught in the wild produced a small number of embryos that showed restricted development before dying. We therefore established a baseline for parthenogenesis in *Drosophila* and specifically in *D. melanogaster*. We used the above parthenogenesis information in different species as a baseline indicator of the degree of parthenogenesis to expect when testing different genotypes of *D. melanogaster* for facultative parthenogenesis. The *D. melanogaster* genotypes were chosen to reflect differential gene expression patterns in mature egg from sexually and parthenogenetically reproducing strains of *Drosophila mercatorum*.

### Preparation Step 2: Temperature selection

We found higher levels of parthenogenesis at lower temperatures in *D. melanogaster*, whereas the very first parthenogenesis screen by Stalker in 1954 using *Drosophila parthenogenetica* found that an increase in temperature increases parthenogenesis.^3^ We therefore suggest performing pilot parthenogenesis assays at different temperatures to determine if there is a temperature effect with the species of interest. This requires one to first identify parthenogenesis in their species of interest. During our preliminary experiments where we screened parthenogenesis as described in this protocol, the first gene variant for which we observed a reproducible amount of parthenogenesis in *D. melanogaster* was the background mutation present in an RNAi line against the gene *bam*. We therefore used this *bam* RNAi to test different temperatures in order to optimize our experiments. Temperature optimization revealed the highest level of parthenogenesis at 18°C, moderate levels at 25°C, and no parthenogenesis at all at 29°C. We chose 25°C for our subsequent experiments as this would allow them to be completed in approximately 3 months compared to 5 months at 18°C due of the difference in lifespan of *D. melanogaster* at these temperatures. Others have also tested keeping eggs at a different temperature to the flies;^3^ this can be done with culture vials after flies have been allowed to lay eggs.

### Preparation Step 3: The selection of appropriate controls

Since parthenogenesis occurs stochastically in *Drosophila*, it is important to have many controls. In addition to negative controls for every experiment, it is also important to examine control gene variants in order to determine if non-specific mutations could cause parthenogenesis under the chosen experimental conditions. In our specific experiments, for example, we had five negative experimental controls: 1) an allele for *CG3436*, a gene that is expressed in the egg and implicated in cell cycle regulation but not differentially expressed, 2) two alleles for *dhd*, another gene that is necessary for the onset of embryogenesis, 3) an allele and an RNAi line for *Klp64D*, a gene that is not involved in the cell cycle and not differentially expressed, 4) an allele and an RNAi line for *Trx-2*, a gene that is necessary for the onset of embryogenesis, and 5) an allele and an RNAi line for *white* (*w*), which was not expressed in the *D. mercatorum* eggs.^1^ There was a low level of parthenogenesis in some of these gene variant controls, indicating that there was a background of parthenogenesis that needed to be accounted for.

In addition to the gene variant controls, we also controlled for other genotypes present in the genetic background. Therefore, since we were using the UAS/Gal4 system to drive expression of interfering RNAs,^8^ we also had controls for the constructs used to drive expression of interfering RNAs and UAS/Gal4. In our case, we tested bag of marbles (*bam*) *gal4*; *maternal tubulin (MTB) gal4* on the 2nd and 3rd chromosomes; and *nanos (nos) gal4*. Both *MTB-gal4* and *nos-gal4 stocks* showed a small degree of parthenogenesis. We also screened the double balancer stock that we used to balance many stocks used in our double variant screen. It too showed a low level of parthenogenesis. Taken together, several of the controls indicated that a considerable number of lab cultured *Drosophila* stocks were capable of extremely low levels of sporadic facultative parthenogenesis.

### Preparation Step 4: Practicalities of parthenogenesis screening

In practical terms, the stock/strain/species should be expanded or amplified prior to starting in order to facilitate the collection of enough virgins for each experiment. Ample fly food is needed for the collection and the maintenance of the flies, and this should be prepared for when running many experiments in parallel. It is also important to note that these experiments are long-running and so it is critical to provide weekly care for the duration of the life of the flies. Some species of *Drosophila* may live 5–6 months at 25°C.

### Virgin collection

**Timing: 2–4 weeks**

1. Collect virgin females.
  a. Remove all males from the vial(s) of enclosing *Drosophila* within every 8 h when at 25°C or every 16 h when at 18°C.
  b. Place females into a fresh vial of food.
  c. Collect virgins and pool until an optimal number of flies per vial is reached.

### Virgin maintenance

**Timing: 1–4 h once a week for 6–22 weeks**

2. Place the vials of virgin female flies at 18°C or at 25°C.
3. Weekly - transfer the virgin females into a new vial with fresh food.
  a. Keep the old food vial at the temperature the flies were maintained at least 3 days (no more than 7).

### Parthenogenesis screening

**Timing: 1–4 h once a week for 6–22 weeks**

4. Screen for offspring after the used food vials have been given sufficient time to allow parthenogenesis to occur.
  a. Examine each vial closely with a dissecting microscope for dead embryos or dead or living larvae.
  b. Keep vials that show evidence of development to ensure that the parthenogens have reached their final stage of development.

## Key resources table

**Table:** 

REAGENT or RESOURCE	SOURCE	IDENTIFIER
**Experimental models: Organisms/strains**		

*D. mercatorum* wild type sexually reproducing	Cornell Stock Center	CSC 15082-1511.00
*D. mercatorum* wild-type facultative parthenogenetic	Cornell Stock Center	CSC 15082-1527.03
*D. mercatorum* parthenogenetic	Cornell Stock Center	CSC 15082-1525.07
*D. ananassae* wild type	Cornell Stock Center	CSC 14024-0371.15
*D. ananassae* wild type	Francis Jiggins Lab	N/A
*D. atripex* wild type	Cornell Stock Center	CSC 14024-0361.00
*D. pallidosa* wild type	Kyorin Fly	k-aae002
*D. suzukii* wild type	Genetics Fly Facility	N/A
*D. parthenogenetica* wild type	Cornell Stock Center	CSC 15181-2221.03
*D. robusta* wild type	Cornell Stock Center	CSC 15020-1111.12
*D. albomicans* wild type	Kyorin Fly	E-10801
*D. americana* wild type	Cornell Stock Center	CSC 15010-1041.32
*D. hydei* wild type	Francis Jiggins Lab	N/A
*S. lebanonensis* wild type	Francis Jiggins Lab	N/A
*S. stonei* wild type	Francis Jiggins Lab	N/A
*D. melanogaster* - w	Genetics Fly Facility	N/A
*D. melanogaster* Or	Genetics Fly Facility	N/A
*D. melanogaster* wild-caught isofemale line – single female collected in Cambridge UK, CB1 1DS	This study	N/A
*D. melanogaster y[1] w[∗]; P{w[+mC] = bam-GAL4:VP16}1*	Bloomington Drosophila Stock Center	BDSC 80579
*D. melanogaster w[∗]; P{w[+mC] = matalpha4-GAL-VP16}V37*	Bloomington Drosophila Stock Center	BDSC 7063
*D. melanogaster w[∗]; P{w[+mC] = matalpha4-GAL-VP16}V2H*	Bloomington Drosophila Stock Center	BDSC 7062
*D. melanogaster Bam-gal4*	Felipe Karam Teixeira Lab	N/A
*D. melanogaster w[1118]; P{w[+mC] = osk-GAL4::VP16}A11/CyO*	Bloomington Drosophila Stock Center	BDSC 44241
*D. melanogaster w[1118]; P{y[+t7.7] w[+mC] = GMR83E05-GAL4}attP2*	Bloomington Drosophila Stock Center	BDSC 40361
*D. melanogaster y[1] w[∗]; P{w[+mC] = Act5C-GAL4}17bFO1/TM6B, Tb[1]*	Bloomington Drosophila Stock Center	BDSC 3954
*D. melanogaster y[1] v[1]; P{y[+t7.7] v[+t1.8] = TRiP.HMC02386}attP2*	Bloomington Drosophila Stock Center	BDSC 44484
*D. melanogaster If/CyO; MKRS/TM6B*	David M Glover Lab	
*D. melanogaster y[1] w[∗]; P{y[+t7.7] = Mae-UAS.6.11}CG3436[LA00324]*	Bloomington Drosophila Stock Center	BDSC 22228
*D. melanogaster dhd[JS]/FM7; TM6B/TM3*	Terry Orr-Weaver Lab	N/A
*D. melanogaster dhd[P8]/FM7*	Terry Orr-Weaver Lab	N/A
*D. melanogaster y[1] v[1]; P{y[+t7.7] v[+t1.8] = TRiP.HMS02193}attP40*	Bloomington Drosophila Stock Center	BDSC 40945
*D. melanogaster y[1] w[1]; Klp64D[k1]/TM3, y[+] Ser[1]*	Bloomington Drosophila Stock Center	BDSC 5578
*D. melanogaster y[1] sc[∗] v[1] sev[21]; P{y[+t7.7] v[+t1.8] = TRiP.HMS00989}attP2*	Bloomington Drosophila Stock Center	BDSC 34019
*D. melanogaster y[1] sc[∗] v[1] sev[21]; P{y[+t7.7] v[+t1.8] = TRiP.HMS00603}attP2*	Bloomington Drosophila Stock Center	BDSC 33721
*D. melanogaster w[1118]; P{w[+mGT] = GT1}Trx-2[BG02804]*	Bloomington Drosophila Stock Center	BDSC 12826
*D. melanogaster* y[1] v[1]; P{y[+t7.7] v[+t1.8] = TRiP.HMS00017}attP2	Bloomington Drosophila Stock Center	BDSC 33623
*D. melanogaster y[1] sc[∗] v[1] sev[21]; P{y[+t7.7] v[+t1.8] = TRiP.GL00094}attP2*	Bloomington Drosophila Stock Center	BDSC 35573
*D. melanogaster y[1] v[1]; P{y[+t7.7] v[+t1.8] = TRiP.HMS00004}attP2/TM3, Sb[1]*	Bloomington Drosophila Stock Center	BDSC 33613
*D. melanogaster y[1] v[1]; P{y[+t7.7] v[+t1.8] = TRiP.HMJ21116}attP40/CyO*	Bloomington Drosophila Stock Center	BDSC 51002
*D. melanogaster y[1] v[1]; P{y[+t7.7] v[+t1.8] = TRiP.HMS00029}attP2*	Bloomington Drosophila Stock Center	BDSC 33631
*D. melanogaster ry[506] e[1] bam[Delta86]/TM3, ry[RK] Sb[1] Ser[1]*	Bloomington Drosophila Stock Center	BDSC 5427
*D. melanogaster y[1] v[1]; P{y[+t7.7] v[+t1.8] = TRiP.HMC03034}attP2*	Bloomington Drosophila Stock Center	BDSC 51428
*D. melanogaster y[1] sc[∗] v[1] sev[21]; P{y[+t7.7] v[+t1.8] = TRiP.HMC04435}attP40*	Bloomington Drosophila Stock Center	BDSC 56993
*D. melanogaster y[1] sc[∗] v[1] sev[21]; P{y[+t7.7] v[+t1.8] = TRiP.HMC03583}attP40*	Bloomington Drosophila Stock Center	BDSC 53354
*D. melanogaster y[1] v[1]; P{y[+t7.7] v[+t1.8] = TRiP.HMJ30095}attP40*	Bloomington Drosophila Stock Center	BDSC 63529
*D. melanogaster y[1] sc[∗] v[1] sev[21]; P{y[+t7.7] v[+t1.8] = TRiP.HMS02251}attP2*	Bloomington Drosophila Stock Center	BDSC 41687
*D. melanogaster y[1] sc[∗] v[1] sev[21]; P{y[+t7.7] v[+t1.8] = TRiP.HMS00249}attP2*	Bloomington Drosophila Stock Center	BDSC 33375

**Other**		

Fly food	https://www.flyfacility.gen.cam.ac.uk/Services/flyfoodservice	Cornmeal food
Dissecting microscope	N/A	N/A
Carbon dioxide, general anesthetic	N/A	N/A
Paintbrush or fly manipulating tool	N/A	N/A

## Materials and equipment

**Table:** Cornmeal Agar Fly Food (makes 140 vials)

Reagent	Final approximate concentration	Amount
Cornmeal (maize)	6.1%	90 g
Yeast	1.8%	26 g
Dextrose	7.1%	105 g
Agar	0.9%	13 g
Nigagin (10% stock)	0.24%	35 mL
Water	N/A	1200 mL
**Total**	**N/A**	**1469 mL**

Note on storage conditions: 18°C–24°C storage, 4 weeks.

## Step-by-step method details

### Virgin collection

**Timing: 2–4 weeks**

Virgin collection from sexually reproducing stocks.

1. Collect virgin females.
  a. Remove all males from the vial(s) of enclosing *Drosophila* within every 8 h when at 25°C or every 16 h when at 18°C. Troubleshooting 1.
  b. Place females into a fresh vial of food.
  c. Collect virgins and pool until an optimal number of flies is reached.

***Note:*** for best results keep the vial of enclosing flies at 25°C during the day and at 18°C during the night.

**CRITICAL:***Drosophila* males can mate with females as soon as their cuticle hardens, therefore it is important to make sure that all males are removed from the vials soon after emergence. In our experiments,^1^ the conditions for virgin collection that we used for the 14 different species was to maintain the vials with enclosing flies at 18°C for maximum of 16 h and at 25°C for a maximum of 8 h.

***Note:*** In our experiments, we minimized substantial age variation in the females being pooled. We noted the collection date as the day the last female was added. Since we expanded our stocks extensively prior to starting virgin collection most were collected and pooled within short time intervals (less than 16 h), the largest age difference between the offspring per pool was 1 calendar day (<48 h difference in age).

**CRITICAL:** It is important to not overpopulate your vials. The flies should all be able to stand at the bottom of the vial, therefore the number depends upon the size of the fly and the size of the vial. In our experiments, we placed 50 *D. melanogaster* females in each vial and 25 *D. mercatorum* females in each vial.

***Note:*** If the flies are highly parthenogenetic then fewer flies should be kept in each vial, we only added 1 female per vial when screening a fully parthenogenetic strain.

***Note:*** It may take several weeks to collect sufficient virgins for an experiment. We recommend collecting 500–1000 females per experiment.

### Virgin maintenance

**Timing: 1–4 h once a week for 6–22 weeks**

Virgin maintenance for the duration of the life of the flies. For the species of *Drosophila* tested by Sperling et al.,^1^ facultative parthenogenesis occurs midway through the life of the fly. Therefore, the flies need to be maintained for several months. The temperature, humidity, and food all need to be controlled during this time.

2. Place the vials of virgin female flies at 18°C or at 25°C.
3. Weekly - transfer the virgin females into a new vial with fresh food. **Note**: the food needs to be changed before it hardens and separates from the side of the vial. Troubleshooting 2.
  a. Keep the old food vial at the temperature the flies were maintained at until two days longer than the time required for normal maximum embryo development at that temperature.

***Note:*** For *D. melanogaster*, the incidence of parthenogenesis decreased with increased temperature^1^ and therefore, an optimal virgin maintenance temperature needs to be selected.

**CRITICAL:** If using a fly incubator, the humidity needs to be maintained to prevent the animals from becoming dehydrated and to prevent the food from drying and hardening.

**CRITICAL:** The fly food needs to be fresh. Many *Drosophilae* will diapause if they have not mated and this effect is exacerbated by not having fresh food.

***Note:*** There is evidence that there is limited variation in the timing of embryogenesis in different *Drosophila* species and for *D. melanogaster* where embryogenesis takes approximately 1 day (24 h).^9^ We suggest adding 2 days to account for differences between species and potential developmental delays that may arise (therefore, 3 days minimum). In practice, we saved the old food vials for 5 days after the virgins were transferred to their new food vial before screening for offspring.

### Parthenogenesis screening

**Timing: 1–4 h once a week for 6–22 weeks**

Screening for the incidence of parthenogenesis in food vials that were used by the virgin females.

4. Screen for offspring after the used food vials have been given sufficient time to allow parthenogenesis to occur.
  a. Examine each vial closely with a dissecting microscope for dead embryos or dead or living larvae. Troubleshooting 3.
  b. Keep vials that show evidence of development to ensure that the parthenogens have reached their final stage of development. Troubleshooting 4 and Troubleshooting 5.

***Note:*** Screening for offspring should be carried out before the vial is more than 7 days post virgin transfer to prevent bacterial or fungal growth from compromising the interpretation of the results.

***Note:*** Dead embryos turn dark brown after cuticle formation during embryonic development (stage 16 of embryogenesis).

## Expected outcomes

**Figure 1:**
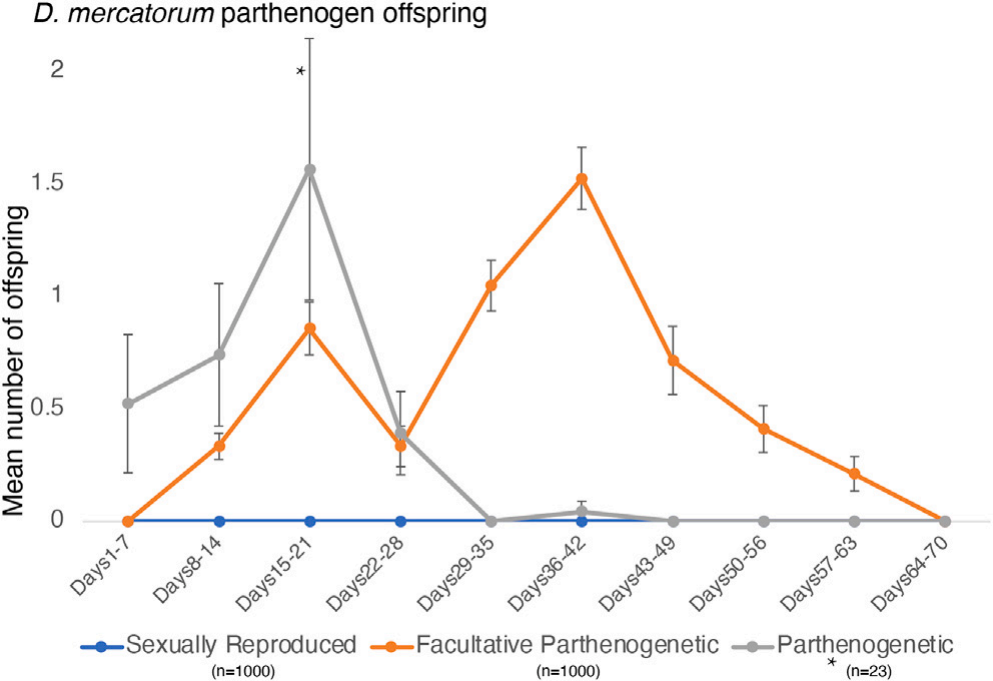
Offspring produced by sexually reproducing, facultative parthenogenetic, and parthenogenetic strains of *D. mercatorum* The mean number of adult offspring produced per week for the entire lifespan of the mothers for sexually reproducing (n = 1000), facultative parthenogenetic (n = 1000), and fully parthenogenetic *D. mercatorum* females (n = 23), adapted from Sperling et al.^1^ The sexually reproducing and facultative parthenogenetic were maintained in batches of 25 and the parthenogenetic were maintained as single flies. Therefore, the parthenogenetic flies are 43× more parthenogenetic than represented in the graph. The error bars represent the standard error.

As the last virgin flies die, tabulate their lifespan and any details about parthenogens produced (Table 1). Most *Drosophila* tested thus far are capable of facultative parthenogenesis,^4^ and it occurs halfway through their lives (Figure 1). Therefore, it is likely that some level of parthenogenesis will be detected. Fully parthenogenetic animals often produce parthenogen offspring early in their life, whereas facultative parthenogens produce offspring midlife (Figure 1).^1^ Facultatively parthenogenetic *Drosophila* will lay many eggs that do not develop.

**Table 1:** Example of parthenogenesis data

Number of flies per vial	Eclosion date for the last female added to the vial	Date the last female died	Maximum lifespan (days)	Parthenogenetic flies	Average age of parthenogenesis	Details
25	11/06/2019	05/08/2019	55	6	29	1 fly at 8 days, 2 flies at 27 days, 2 flies at 34 days, 1 fly at 41 days
25	13/06/2019	05/08/2019	53	7	33	3 flies at 25 days, 1 fly at 34 day, 3 flies at 41 days
25	27/06/2019	16/08/2019	50	1	43	1 fly at 43 days

## Quantification and statistical analysis

Statistical analysis should be performed using Fisher’s exact test, example in R below, since it allows for low numbers of positive cases or a chi-squared test if there are higher incidences of positive cases.

>TBL = rbind(c(sample-positives, sample-negatives), c(control-positives, control-negatives)); TBL

>fisher.test(TBL)

#or

>chisq.test(TBL)

## Limitations

The limitation to this protocol is following the requirement of waiting until the flies die until the experiment is done, this can be time consuming. Furthermore, the food being changed and screened weekly limits what time of year these types of experiments should be initiated, for example it is best not to start them before having a prolonged period of time without going to the laboratory. Missing food changes or having delayed screening can make the results unreliable.

## Troubleshooting

### Problem 1

Step 1a, mated females: Consistently finding that the females collected were not virgins.

### Potential solution

When testing the parthenogenetic ability of a new species, we recommend performing preliminary assays to determine the time needed for the males to be able to mate by taking freshly eclosed males and giving them access to virgin females over a time course. There is variation in reproductive maturity in *Drosophila* males.^10^ Therefore, preliminary testing will give an indication of how often it is necessary to collect virgins.

### Problem 2

Step 3, Dry food. Finding that the food pulls away from the vial and the *Drosophila* get stuck.

### Potential solution

Adjust the humidity of the incubator or regularly mist the vials if the humidity cannot be controlled by the incubator. The humidity of the incubator should be maintained between 50-78% (the upper range being ideal).

### Problem 3

Step 4a, Embedded eggs. Some *Drosophilae*, such as *Drosophila suzukii* and *D. mercatorum*, embed their eggs into the food and the eggs may be difficult to see if they are deep within the food.

### Potential solution

High magnification, careful attention, and illumination of the vial from the side is needed, making the screening more time-consuming.

### Problem 4

Step 4b, Surprise male offspring. Drosophilae have a spermatheca that can hold hundreds of sperm for long periods of time.^11^^,^^12^ If they have mated it will become obvious by the presence of many male and female offspring. However, because *Drosophila* produce offspring with male characteristics as a result of nondisjunction (X/0 males) during meiosis,^4^ the presence of males does not necessarily mean that the mothers are not virgin.

### Potential solution

If in doubt, then the X/0 male can be back crossed to virgin females and checked for offspring. If no offspring emerge in the time when a control male can produce offspring, then it may be a sterile male fly that is lacking the Y chromosome.

### Problem 5

Step 4b, Stochastic Parthenogenesis in lab stocks. If screening *D. melanogaster,* it is important to keep in mind that many lab stocks show very low levels of stochastic parthenogenesis.^1^ In addition to the sporadic facultative parthenogenesis seen in the controls, we found that the many RNAi lines used in the primary screen had a small degree of parthenogenesis without expression being driven by Gal4. These were the lines for *bam*, *CG4496*, *CG10433*, *CRMP*, *Desat2*, *f*, and *FER*, and for the controls *Klp64D* and *w*. In all these cases, the RNAi line had fewer instances of parthenogenesis when crossed to the Gal4 drivers, and thus when the expression of the RNAi was induced. This indicates that the RNAi does not itself cause parthenogenesis but rather it is the result of something in the background of the RNAi stocks.

### Potential solution

We tested the notion of a background mutation in the RNAi lines using the *bam* RNAi line. We crossed this line to 8 different gal4 lines (*bam* (X), *bam* (III), *nos*, MTB (II), MTB (III), *actin* (act), and *ovarian tumor* (*otu*)) and found that some could drive a low level (0.1–0.2%) of parthenogenesis but at levels less than or equal to the RNAi stock alone (0.2%). We also crossed the RNAi line to the *white* mutant allele that did not have any degree of parthenogenesis on its own and found that there was still 0.2% parthenogenesis. Finally, we checked that the observed parthenogenesis could not be caused by the RNAi target gene by crossing the *bam* RNAi line to a mutant allele of *bam* and found that this reduced the level of parthenogenesis to 0.1%. Together, the evidence strongly suggests that the low degree of parthenogenesis is caused by a background mutation: (1) It is not the presence of the RNAi construct since other RNAi lines did not show any parthenogenesis, (2) It is not the expression of the RNAi since driving the expression did not enhance the degree of parthenogenesis. (3) It is not due to the *bam* mutation because crossing the RNAi line to a *bam* null mutant allele did not enhance the degree of parthenogenesis. The cause of stochastic parthenogenesis is likely due to the presence of the *Desat2* allele which is present in most *Drosophila* stocks.^13^ The variation in the ability of the *Desat2* allele to result in parthenogenesis is likely a consequence of the genetic background of the stock or strain, which has been found to influence the desaturase activity.^14^ Therefore, if a high background of parthenogenesis is found when doing a gene variant screen one can follow the above method to identify if it is the gene in question.

## Resource availability

### Lead contact

Further information and requests for resources and reagents should be directed to and will be fulfilled by the lead contact, Alexis Sperling (alb84@cam.ac.uk).

### Materials availability

This study did not generate new unique materials or reagents.

## Data Availability

This study did not generate/analyze datasets/code.

## References

[1] Sperling A.L., Fabian D.K., Garrison E., Glover D.M. (2023). A genetic basis for facultative parthenogenesis in Drosophila. Curr. Biol..

[2] Markow T.A. (2013). Parents Without Partners: Drosophila as a Model for Understanding the Mechanisms and Evolution of Parthenogenesis. G3 (Bethesda).

[3] Stalker H.D. (1954). Parthenogenesis in Drosophila. Genetics.

[4] Sperling A.L., Glover D.M. (2023). Parthenogenesis in dipterans: a genetic perspective. Proc. Biol. Sci..

[5] Stalker H.D. (1964). The salivary gland chromosomes of *Drosophila nigromelanica*. Genetics.

[6] Templeton A.R., Carson H.L., Sing C.F. (1976). The population genetics of parthenogenetic strains of *Drosophila mercatorium*. II The capacity for parthenogenesis in a natural, bisexual population. Genetics.

[7] Carson H.L. (1967). Selection for parthenogenesis in *Drosophila mercatorum*. Genetics.

[8] Brand A.H., Perrimon N. (1993). Targeted gene expression as a means of altering cell fates and generating dominant phenotypes. Development.

[9] Kuntz S.G., Eisen M.B. (2014). Drosophila Embryogenesis Scales Uniformly across Temperature in Developmentally Diverse Species. PLoS Genet..

[10] Pitnick S., Markow T.A., Spicer G.S. (1995). Delayed male maturity is a cost of producing large sperm inDrosophila. Proc. Natl. Acad. Sci. USA.

[11] Neubaum D.M., Wolfner M.F. (1999). Wise, winsome, or weird? Mechanisms of sperm storage in female animals. Curr. Top. Dev. Biol..

[12] Dhillon A., Chowdhury T., Morbey Y.E., Moehring A.J. (2020). Reproductive consequences of an extra long-term sperm storage organ. BMC Evol. Biol..

[13] Dallerac R., Labeur C., Jallon J.-M., Knipple D.C., Roelofs W.L., Wicker-Thomas C. (2000). A D9 desaturase gene with a different substrate specificity is responsible for the cuticular diene hydrocarbon polymorphism in *Drosophila melanogaster*. Proc. Natl. Acad. Sci. USA.

[14] Dembeck L.M., Böröczky K., Huang W., Schal C., Anholt R.R.H., Mackay T.F.C. (2015). Genetic architecture of natural variation in cuticular hydrocarbon composition in Drosophila melanogaster. Elife.

